# Step-by-step: A clinical pathway for stepped care management of fear of cancer recurrence—results of a three-round online delphi consensus process with Australian health professionals and researchers

**DOI:** 10.1007/s11764-024-01685-1

**Published:** 2024-10-07

**Authors:** Allan ‘Ben’ Smith, Afaf Girgis, Natalie Taylor, Alison Pearce, Jia Liu, Heather L. Shepherd, Verena S Wu, Gail Garvey, Laura Kirsten, Iman Zakhary, Carolyn Ee, Daniel Ewald, Annie Miller, Joanne Shaw

**Affiliations:** 1 The Daffodil Centre, The University of Sydney, A Joint Venture with Cancer Council NSW, Sydney, NSW Australia; 2 South West Sydney Clinical Campuses, UNSW Medicine & Health, UNSW Sydney, Liverpool, NSW Australia; 3 School of Population Health, UNSW Sydney, Sydney, NSW Australia; 4 Sydney School of Public Health, The University of Sydney, Sydney, NSW Australia; 5 St Vincent’s Hospital, Sydney, NSW Australia; 6 UNSW Medicine & Health, UNSW Sydney, Sydney, NSW Australia; 7 Garvan Institute of Medical Research, Sydney, NSW Australia; 8 Susan Wakil School of Nursing and Midwifery, Faculty of Medicine and Health, The University of Sydney, Sydney, NSW Australia; 9 The School of Public Health, Faculty of Medicine, The University of Queensland, Brisbane, QLD Australia; 10 Nepean Cancer Services, Nepean Blue Mountains Local Health District, Penrith, NSW Australia; 11 School of Psychology, Psycho-Oncology Cooperative Research Group, The University of Sydney, Sydney, NSW Australia; 12 Multicultural Services, South Western Sydney Local Health District, Liverpool, NSW Australia; 13 Caring Futures Institute, Flinders University, Bedford Park, SA Australia; 14 NICM Health Research Institute, Western Sydney University, Penrith, NSW Australia; 15 Lennox Head Medical Centre, Lennox Head, NSW Australia; 16 Bullinah Aboriginal Health Service, Ballina, NSW Australia; 17 Sydney University Medical School, Northern Rivers University Centre for Rural Health, Lismore, NSW Australia; 18 Cancer Council NSW, Woolloomooloo, NSW Australia

**Keywords:** Fear of cancer recurrence, Oncology, Clinical pathway, Cancer survivorship, Screening, Stepped care

## Abstract

**Purpose:**

Fear of cancer recurrence (FCR) is not routinely addressed in clinical practice, meaning many cancer survivors forego effective interventions. We established expert consensus on a clinical pathway to help health professionals identify and manage FCR in early-stage cancer survivors.

**Methods:**

Australian health professionals and researchers working with adult cancer survivors participated in a three-round Delphi study promoted via oncology professional bodies and social media. The Round 1 online survey presented 38 items regarding FCR screening, triage, assessment, referral, and stepped care, based on a literature review, related pathways/guidelines, and expert input. Participants rated how representative of best-practice items were on a 5-point scale (strongly disagree–strongly agree), with optional qualitative feedback. Consensus was defined as ≥ 80% of participants strongly/agreeing with items. Items not reaching consensus were re-presented to Round 1 participants in two subsequent rounds with new items, derived from content analysis of qualitative feedback.

**Results:**

From 94 participants in Round 1 (89% health professionals), 26/38 (68%) items reached consensus. By round 3, 35/38 (92%) items, including 8 new items, reached consensus. Routine FCR screening and triage conversations and stepped care management (i.e. tailored and staged treatment) were endorsed. However, the timing of FCR screening/triage did not reach consensus.

**Conclusions:**

This world-first FCR clinical pathway incorporating contemporary evidence and expert opinion recommends routine screening and triage to stepped care management of FCR. Some pathway components, such as screening or triage timing, may need tailoring for different contexts.

**Implications for Cancer Survivors:**

Implementation of the pathway could aid routine identification and management of FCR, reducing its burden on cancer survivors and the healthcare system.

**Supplementary Information:**

The online version contains supplementary material available at 10.1007/s11764-024-01685-1.

## Introduction

There are a growing number of people living with and beyond cancer (i.e. cancer survivors) worldwide. Most cancer survivors experience some degree of fear, worry, or concern about their cancer coming back or getting worse, referred to as fear of cancer recurrence (FCR) [1]. About 40% experience moderate FCR, while almost one in five (19.2%) experience severe/clinical FCR [2], characterised by persistently high levels of preoccupation and worry, hypervigilance to physical symptoms, and functional impairment [3]. Poorer mental health and quality of life [4] and greater healthcare use [5] are related to greater FCR severity.

Despite its prevalence, lack of routine screening means FCR may often be missed in routine practice. Many cancer survivors are reluctant to raise FCR with health professionals, as they do not want to seem ungrateful for their treatment or to be questioning its efficacy [6]. Health professionals may hesitate to ask about FCR, due to worry about inducing concerns in their patients [6], and may otherwise struggle to identify patients with FCR, as it is not closely related to clinical factors, such as cancer stage or treatment [2]. To address this gap, FCR screening and assessment tools have been developed and validated [7], but they are not routinely used in practice to identify FCR and guide management.

There are effective interventions for FCR [8], largely originating from North America (e.g. FORT [9]), Europe (e.g. SWORD [10]), and Australasia (e.g. ConquerFear [11]), but implementation in practice is a challenge [12]. Barriers include the fact that most FCR interventions lack referral pathways, are time and resource-intensive, are delivered by specialist psychologists, and have largely been evaluated in English-speaking breast cancer survivors [8, 13]. This can limit implementation among more diverse populations, such as minority groups, those living in regional/rural areas, and those with less specialist resources. Many survivors may not need specialist-delivered interventions for FCR, but guidance to aid referral to appropriately intense FCR intervention has been limited. Canadian FCR guidelines were published in 2024 [14], but specific recommendations regarding how to integrate existing FCR screening and assessment tools in clinical practice to identify those with FCR and guide appropriate treatment are lacking [15]. Consequently, most health professionals report challenges managing FCR [16], and survivors as well as carers report FCR as their greatest unmet supportive care need post-treatment [17].

To address this evidence-practice gap, research focused on enhancing FCR intervention access and implementation in practice has been identified as an Australian [18] and international research priority [19]. Clinical pathways offer a promising avenue for translating evidence into practice, by providing standardised, evidence-based, and context-specific recommendations for multidisciplinary care, which have been shown to facilitate the implementation of evidence in clinical practice [20]. The Delphi technique, which comprises an iterative series of anonymous surveys of experts with feedback after each round, has previously been used to establish consensus regarding clinical pathway elements [21, 22]. Seeking consensus from experts, such as health professionals, who often need to consider how to optimally apply evidence in a broader range of populations and contexts to those in which it is originally collected, can help overcome evidence limitations including lack of diversity among research participants.

We aimed to use the Delphi technique to obtain expert consensus on clinical pathway elements (i.e. recommended activities) relating to three key components of addressing FCR: (1) screening; (2) triage, assessment, and referral; and (3) treatment, in the context of routine clinical care of early-stage cancer survivors, the population in which most FCR research has been conducted to date. Our primary objective was to obtain expert consensus regarding what clinical pathway elements were optimal, that is, representative of best practice and likely to achieve the best outcome for cancer survivors. Our secondary objective was to seek consensus on how feasible (i.e. able to be implemented in practice) clinical pathway elements were. The feasibility of clinical pathway elements and potential strategies to facilitate implementation will be reported separately.

## Methods

Ethical approval for a three-round Delphi study seeking expert consensus on elements of a clinical pathway for FCR was obtained from the University of New South Wales Human Research Ethics Committee (HC230052) and undertaken in accordance with Conducting and REporting Delphi Studies (CREDES) recommendations [23].

### Draft clinical pathway

Prior to the Delphi study, a draft clinical pathway was developed based on the following:

1. A related Australian clinical pathway for anxiety and depression in adult cancer patients [22]. Considering that the anxiety and depression and FCR clinical pathways target intersecting forms of psychological distress experienced by adult cancer survivors in the Australian healthcare context and may be implemented in tandem, it was anticipated that elements of the two clinical pathways would be similar. Informal process mapping of current practices around FCR identification and management confirmed this. Consequently, the draft FCR clinical pathway adopted the same overall components as the anxiety and depression clinical pathway (i.e. screening, assessment, stepped care).
2. A literature review of both academic and grey literature informed specific FCR clinical pathway elements. In 2014, Cancer Australia published a guideline for the identification and management of FCR in adult cancer survivors [47] incorporating evidence up to 2012. A scoping review of literature regarding the effectiveness of FCR screening, assessment, and treatment was conducted from 2012 to January 2023. Evidence from systematic reviews was prioritised, but original research was considered if there were no systematic reviews.
3. Draft clinical pathway elements were presented to an expert panel comprising consumers, researchers, and clinicians with expertise in FCR from multiple disciplines (i.e. psychology, nursing, medical oncology, implementation science, health economics, health services, Indigenous health) for review and feedback.

At the end of this process the draft clinical pathway comprised six key components with varying numbers of elements, each represented by a Delphi study item: (1) screening (7 elements); (2) triage, assessment and referral (5 elements); (3) post-screening/triage stepped care (8 elements); (4) universal care for minimal to mild FCR (5 elements); (5) supported self-management for moderate FCR (6 elements); (6) specialist care for severe FCR (5 elements).

The draft clinical pathway was broadly consistent with Canadian FCR guidelines published in 2024 [14]. Both recommend FCR screening, assessment, and treatment tailored to FCR severity, but the guidelines suggest a matched care approach, while the pathway proposed stepped care. The draft clinical pathway aimed to provide further detail regarding who, when, and how recommended activities were carried out to aid implementation in practice.

### Delphi process

The draft clinical pathway was presented in the initial online Delphi survey created using Qualtrics. The survey was iteratively developed with feedback from the expert panel and then piloted with two clinical experts in cancer survivorship (Prof Michael Jefford and Prof Bogda Koczwara). This resulted in the addition of items focused on managing FCR in primary care and item wording refinement. An online participant information sheet, consent form, and demographic items were presented prior to the Delphi items, which were grouped according to pathway components (e.g. FCR screening). Each survey section included a description and summary of the evidence regarding a particular component (see Supplementary File 1 for example). For the stepped care components, figures outlining the proposed stepped care model and key elements of each step were provided.

Delphi items were generally presented in the form of a statement that something ‘should’ occur or is necessary (e.g. ‘Specific clinical staff should be designated to review the results of FCR screening’). Participants were asked to indicate their level of agreement about how optimal (i.e. representative of best practice and likely to achieve the best outcomes for cancer survivors) each statement was on a 5-point Likert scale ranging from ‘strongly disagree’ to ‘strongly agree’. The definition of optimal was repeated on each survey page. Participants could select ‘Not my expertise’ if they did not feel qualified to rate a particular item. Participants could also provide comments in a free text box next to each item or in a question at the end of the section for each component.

Consensus was defined as ≥ 80% of participants agreeing/strongly agreeing an item was optimal, in which case it was retained, or ≥ 80% of participants disagreeing/strongly disagreeing an item was optimal, in which case it was discarded. Round 2 and 3 surveys included an overview of how many items reached consensus across each clinical pathway component in the previous round and a list of items that reached consensus. For items that *did not reach consensus* in the previous round, a summary of quantitative ratings and content analysis of qualitative feedback was presented. Participants were asked to rate how optimal the original item and a revised (i.e. new) item based on content analysis of qualitative feedback were, while considering the summary of previous round responses. If the revised item reached consensus, but the original item did not, the revised item was retained, and the original item was discarded. The process was continued until all items (both original and revised) not reaching consensus had been (re-)presented at least once. Items approaching consensus (approx. 70 to 80% agreement) after three survey rounds were discussed by the expert panel.

The initial survey was distributed by oncology professional bodies (see Acknowledgements) and promoted via presentations and social media. Participants provided their email addresses during study consent, but emails were not linked to Delphi item responses to ensure anonymity. Subsequent surveys were emailed to all participants who consented to the Round 1 survey. No new participants were recruited; Round 2 and 3 participants were a subset of the Round 1 sample. All Round 2 and 3 participants were presented with the same items that did not reach consensus from the previous round (see Table 2). Three reminders were sent for each survey round.

### Participants

Eligible participants were health professionals from any discipline currently working with adult patients with cancer, or researchers who had co-authored a peer-reviewed article on FCR in the last 5 years, able to complete the online survey in English, and willing to provide an email address for subsequent survey rounds. We did not deliberately sample health professionals working with patients of any particular cancer site, stage, treatment phase, or survivorship duration. Collaborators of the study investigators were not directly solicited to take part but were not excluded. People working in paediatric oncology or outside Australia were excluded, as contextual differences may have impacted item ratings.

There are no concrete guidelines for the optimal number of Delphi study participants to achieve consensus, but a sample of 12 participants is generally considered to be the minimum required if the background/discipline of participants is homogenous [24]. As addressing FCR is a multidisciplinary exercise, we aimed to recruit 48 participants, with ideally 12 participants from each of the following groups: (1) mental/allied health, (2) physicians, (3) nurses, (4) researchers (i.e. 75% clinicians, 25% researchers). We aimed to include 12 or more participants from each background, to maximise the likelihood that pooled judgements of study participants were representative of their discipline, enabling comparison of consensus levels by discipline if required for items approaching consensus. A greater proportion of clinicians was sampled, considering their unique perspective on applying the clinical pathway in practice, but all responses were weighted equally. Allowing for a 30% initial response rate, as per a Delphi study establishing a similar clinical pathway [22], and 20% attrition in subsequent survey rounds, we aimed to approach at least 200 eligible participants (≥ 50 from each group). Considering the professional bodies that distributed the survey (see Acknowledgements for list) have hundreds of members, it is likely this target was exceeded.

### Analysis

Descriptive statistics were used to determine levels of consensus according to the criteria above. Percentage calculations excluded participants with missing data or answering ‘Not my expertise’ for an item. For items not reaching consensus, content analysis of open-ended responses was conducted by two experienced qualitative researchers (ABS and VSW). Each unit of text (i.e. piece of feedback) was coded inductively by VSW, and related codes were then grouped into categories for each item [25], which were reviewed by ABS and refined through discussion with VSW until consensus was reached. For items approaching consensus after the third survey round, two-tailed Kruskal-Wallis exact tests were used to assess inter-discipline differences and inform discussion regarding inclusion by the expert panel. Analyses were conducted using SPSS Statistics Version 28.0 (IBM Corp); *p* values < .05 were considered significant.

## Results

Ninety-four participants consented and completed the demographics survey, of which 89 completed the first round (April-June 2023), 69 completed the second round (July-August 2023), and 73 completed the third round (November-December 2023) of the Delphi survey. As the survey was distributed on behalf of the research team by oncology professional bodies, the response rate of potentially eligible participants is unknown. As participant emails and other personal/demographic characteristics were not linked to Delphi survey responses to ensure anonymity, the specific characteristics of round 2 and 3 respondents are unknown. Consenting participants were primarily (86%) health professionals and came from multidisciplinary backgrounds: 31 (33%) mental/allied health (18 psychology, 13 social work), 30 (32%) nursing, 18 (19%) medical/radiation/surgical oncology (Table 1).

**Table 1:** Sample characteristics (*N* = 94) of participants who gave consent and completed the demographics survey

Variable		*N* (%) of respondents
Age	21–30	3 (3.2)
	31–40	25 (26.6)
	41–50	28 (29.8)
	51–60	24 (25.5)
	61–70	13 (13.8)
	Prefer not to say	1 (1.1)
Gender	Man/male	10 (10.6)
	Woman/female	84 (89.4)
Country of birth	Australia	71 (75.5)
	Other	23 (24.5)
Language spoken at home	English	82 (87.2)
	Other	12 (12.8)
Aboriginal/Torres Strait Islander status	Non-Indigenous	94 (100)
Primary role	Clinician	81 (86.2)
	Researcher	10 (10.6)
	Educator	3 (3.2)
Primary focus	Psychology	18 (19.1)
	Social work	13 (13.8)
	Nursing	30 (31.9)
	Medical oncology	12 (12.8)
	Radiation oncology	5 (5.3)
	Surgical oncology	1 (1.1)
	Palliative care	1 (1.1)
	Other	14 (14.9)
Work setting (clinician)^a^	Tertiary referral cancer centre	44 (46.8)
	District/local hospital	22 (23.4)
	Non-inpatient cancer treatment centre	18 (19.1)
	Non-hospital based	10 (10.6)
	Primary care	2 (2.1)
	Community centre	6 (6.4)
	Other	6 (6.4)
Public/private (clinician)^a^	Public	51 (54.3)
	Private	7 (7.4)
	Public and private	17 (18.1)
	Public charitable organisation	3 (3.2)
	Private charitable organisation	5 (5.3)
	Other	2 (2.1)
Years worked with cancer survivors (clinician)	0–2	3 (3.2)
	3–5	11 (11.7)
	6–10	22 (23.4)
	10+	48 (51.1)
Role caring for cancer survivors with FCR (clinician)^a^	Screening and/or assessment	49 (52.1)
	Referral to psychosocial support	55 (58.5)
	Provision of psychosocial support	47 (50)
Years conducting research (researcher)	6–10	3 (3.2)
	10+	7 (7.4)

^a^Respondents could select multiple responses

**Fig. 1:**
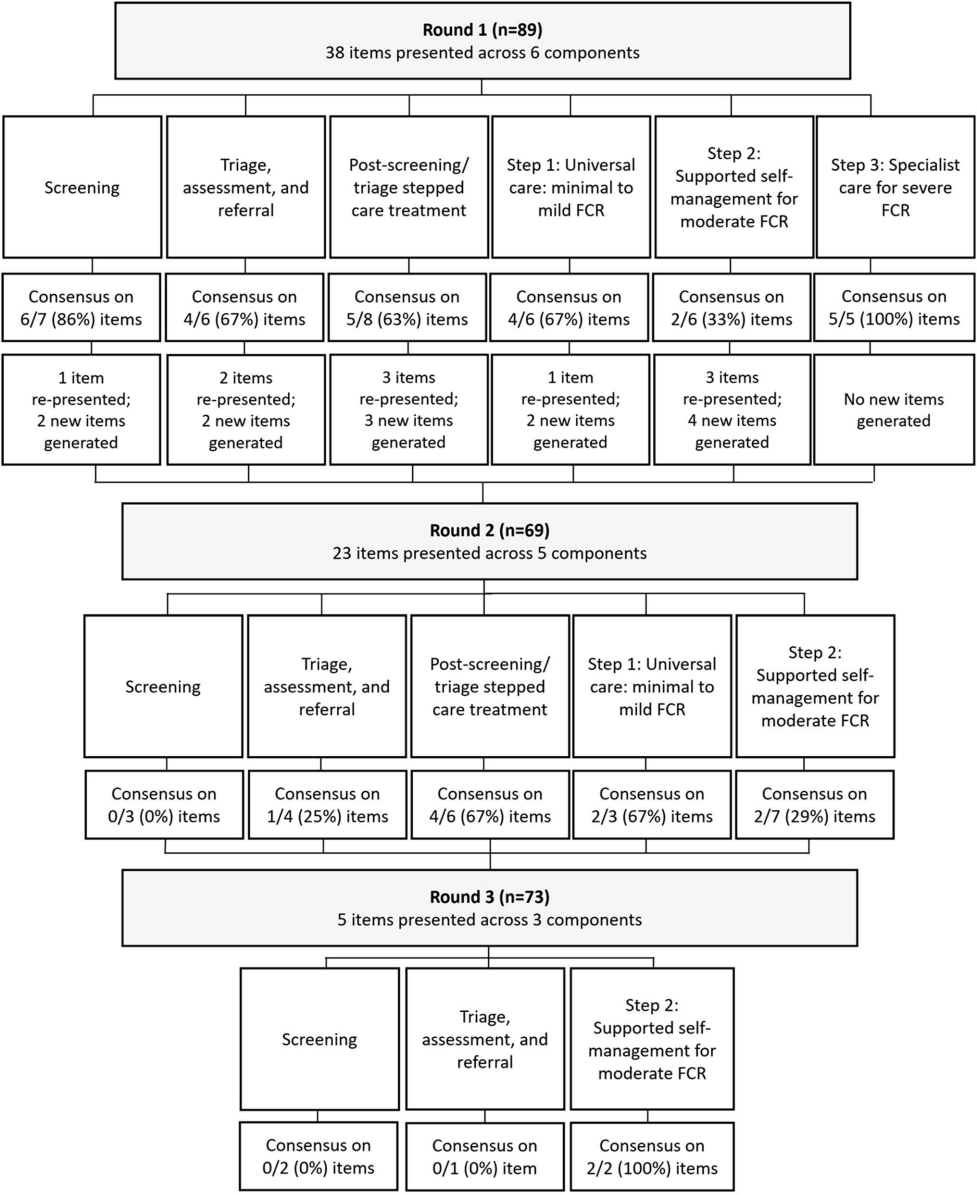
Delphi consensus process

Figure 1 shows the consensus process. In Round 1, 38 items were presented, and 26 (68%) reached consensus. In Round 2, 23 (10 original, 13 new) items were presented, and 9 (39%) reached consensus. In Round 3, 5 items were presented, 2 (40%) reached consensus, and 3 approached consensus and were discussed by the expert panel. All items reached consensus for being retained (≥ 80% agreement), as opposed to being discarded (≥ 80% disagreement). The number and proportion of participants responding ‘not my expertise’ ranged from 1 (1.2%) for item 14 (‘A stepped care model is appropriate for managing FCR in cancer survivors’) to 3 (3.8%) for item 29a (‘Moderate FCR should primarily be addressed outside the hospital setting’). Due to the low frequency of ‘not my expertise’ responses, differences according to group (mental/allied health vs nurse vs physician vs researcher) could not be calculated. Content analysis of qualitative feedback on items that did not initially reach consensus is presented in Supplementary File 2.

**Fig. 2:**
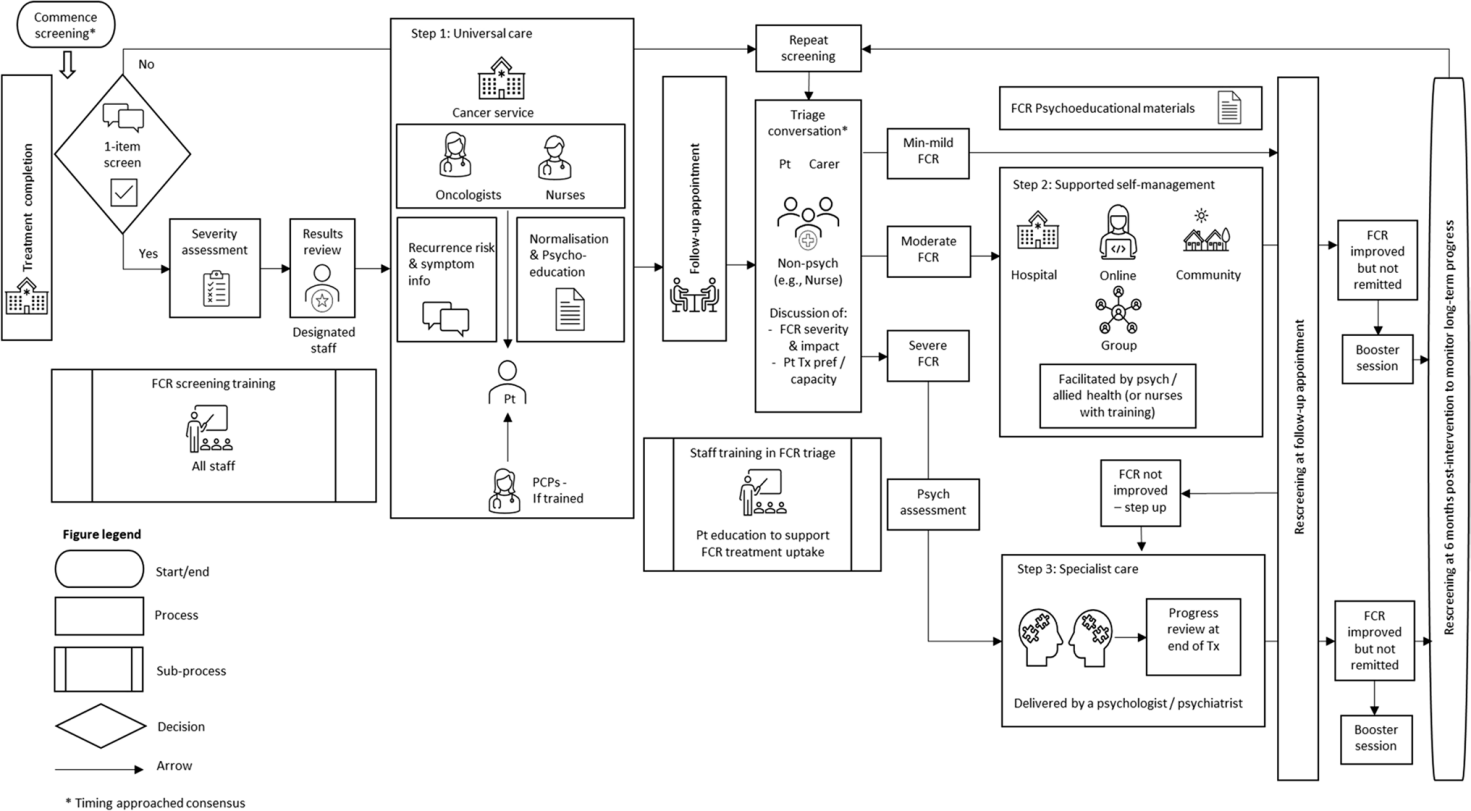
Overview of the FCR clinical pathway

Table 2 details the results of each survey round. Results for each clinical pathway component after three Delphi survey rounds are summarised under the headings below. The final six-component consensus-based clinical pathway for the optimal identification and management of FCR can be seen in Figure 2.

**Table 2:**
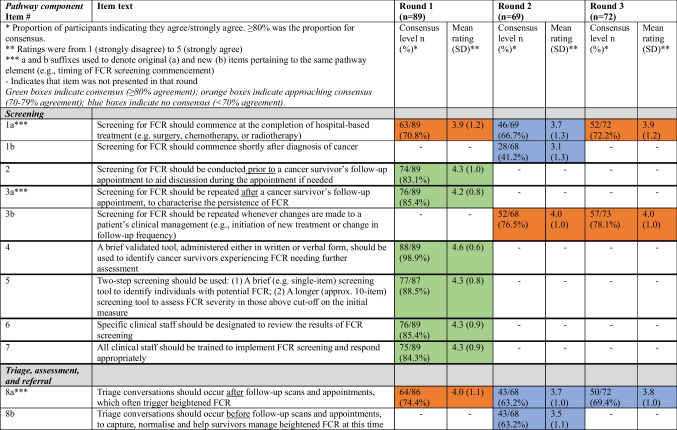

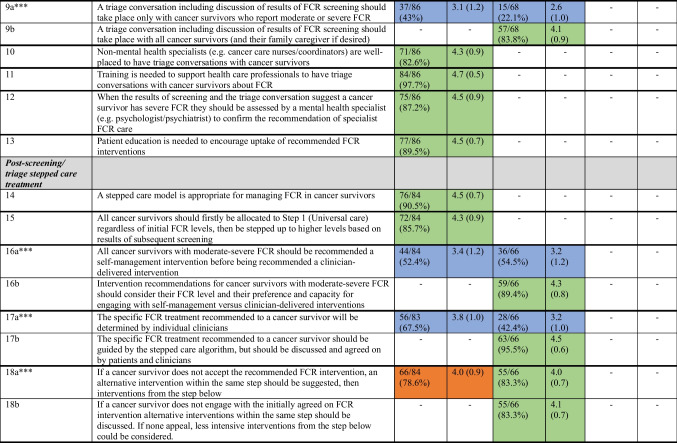

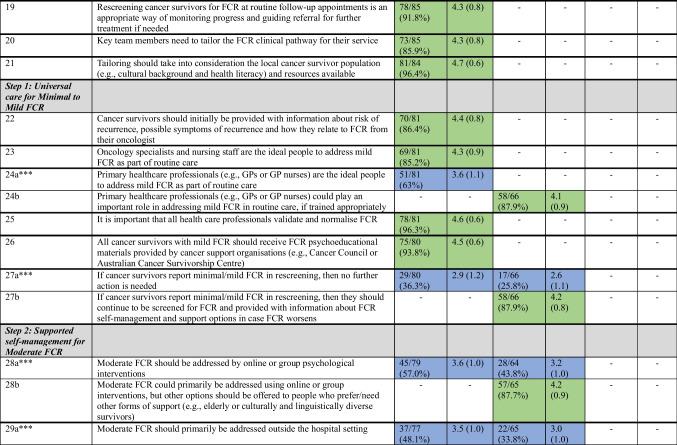

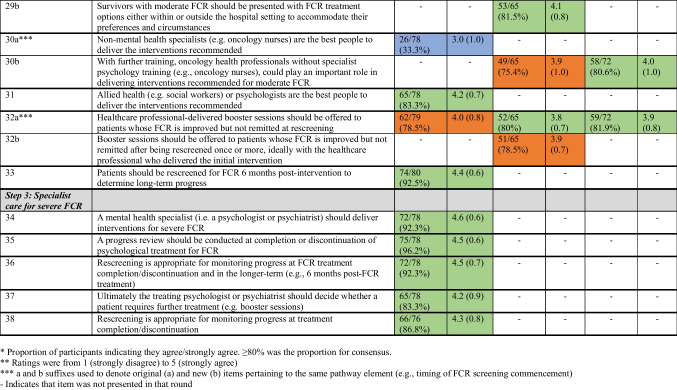
Consensus levels for items presented according to round

*Proportion of participants indicating they agree/strongly agree. ≥ 80% was the proportion for consensus

**Ratings were from 1 (strongly disagree) to 5 (strongly agree)

***a and b suffixes used to denote original (a) and new (b) items pertaining to the same pathway element (e.g. timing of FCR screening commencement)

-Indicates that the item was not presented in that round

### Screening

Consensus was reached on six of eight proposed clinical pathway elements. A two-step process, comprising the use of a brief validated tool to identify people with potential FCR, who would then be administered a slightly longer measure to assess severity was endorsed. It was agreed that FCR screening should be conducted both before and after follow-up appointments to aid FCR discussion during appointments and characterise the persistence of FCR, respectively. There was consensus that designated clinical staff should review screening results, but all clinical staff should be trained to implement FCR screening and respond appropriately.

Consensus was not reached regarding when to start FCR screening. Approximately 70% of participants in each round agreed it should be at the completion of hospital-based treatment, while 41% of Round 2 participants agreed it should be shortly after diagnosis. There were no differences in Round 3 agreement ratings regarding starting screening at the completion of hospital-based treatment according to group (mental/allied health vs nurse vs physician vs researcher) (*χ*^2^(3) = 1.82, *p* = 0.61). The recommendation that FCR screening should be repeated whenever changes are made to a patient’s clinical management (e.g. change in treatment or follow-up) also approached consensus (78% agreement). There were no differences in Round 3 agreement ratings according to group (*χ*^2^(3) = 4.53, *p* = 0.21). After consideration by the expert panel, it was decided to recommend that FCR screening start at the completion of hospital-based treatment, as uncertainty around when to start screening could lead to missed screening, but not dictate when FCR screening should be repeated in relation to clinical management changes, as these could be for varied reasons.

### Triage, assessment, and referral

Consensus was reached on five of six proposed clinical pathway elements. It was agreed that triage conversations should take place with *ALL* cancer survivors and family caregivers (if desired). The need for health professional training to enable these conversations and patient education to encourage uptake of recommended FCR interventions were supported. Non-mental health specialists (e.g. cancer care nurses/coordinators) were endorsed as well-placed to have triage conversations with cancer survivors, with indications of severe FCR to be confirmed by mental health specialist assessment.

Consensus was not reached (69% agreement) regarding whether triage conversations should occur before or after follow-up scans and appointments. There were no differences in Round 3 agreement ratings according to group (*χ*^2^(3) = 1.05, *p* = 0.79). After discussion by the expert panel, it was decided not to recommend specific timing for triage conversations, noting that this may be determined by FCR severity reported in screening and service characteristics.

### Post-screening/triage stepped care treatment

Consensus was reached on all eight proposed clinical pathway elements. It was agreed that all cancer survivors should initially receive universal care and then be stepped up based on FCR severity reported in subsequent screening. There was consensus that FCR treatment should be guided by the stepped care algorithm, alongside discussion of patients’ preferences/capacity to engage with recommended interventions, and presentation of alternative interventions of similar intensity if needed. It was also agreed that key team members should tailor the clinical pathway to their local population and resource availability.

### Step 1: Universal care for minimal to mild FCR

Consensus was reached on all six proposed clinical pathway elements. The importance of all health professionals validating and normalising FCR was endorsed. It was agreed that cancer survivors reporting mild FCR should receive FCR psychoeducational materials and information about recurrence risk and symptoms from their oncologist and continue to be screened in case FCR worsens. There was consensus that oncology specialists and nursing staff are the ideal people to address mild FCR as part of routine care, but that primary care practitioners could play an important role, if trained appropriately.

### Step 2: Supported self-management for moderate FCR

Only two of six proposed clinical pathway elements reached consensus after Round 1, although all six ultimately reached consensus. It was agreed that survivors with moderate FCR should be offered a choice of interventions (primarily online or group), delivered within or outside the hospital setting, to accommodate varied preferences and circumstances. Allied health (e.g. social workers) or psychologists were initially endorsed as best placed to deliver recommended interventions. However, in Round 3, it was agreed that with further training, non-mental health specialists (e.g. oncologists/nurses) could also play a role. There was support for FCR rescreening at 6 months post-intervention, with health professional-delivered booster sessions offered to patients whose FCR is improved but not remitted.

### Step 3: Specialist care for severe FCR

All five proposed clinical pathway elements reached consensus after Round 1. It was agreed that a mental health specialist should deliver interventions for severe FCR, a progress review should be completed at discontinuation/completion of FCR treatment, rescreening could be used for monitoring longer-term progress, and the treating psychologists/psychiatrist should ultimately decide if further treatment was required.

## Discussion

This study has produced the first consensus-based clinical pathway for the optimal identification and management of FCR according to Australian health professionals and researchers working with adult cancer survivors. After three Delphi survey rounds there was agreement on most proposed elements across the key clinical pathway components (I.e., screening; triage, assessment, and referral; and stepped care management). The optimal timing of FCR screening and triage did not reach consensus.

Screening for FCR is fundamental to the clinical pathway and is particularly important considering that many patients may be reluctant to raise FCR [6]. There was near-universal agreement that a brief screening tool should be used for this purpose. Suitable validated tools include the verbally administered FCR-1 [26] or the visually presented FCR-1r [27], which is recommended by Canadian FCR guidelines [14]. The Distress Thermometer and problem list, which is commonly used in the USA [28] and Europe [29], does not appear suitable for FCR screening [30]. Embedding screening tools in patient-reported outcome measures (PROMs) systems and integrating these into clinical workflows and care pathways are priority implementation strategies [31]. The FCR-1r was designed to be incorporated into the Edmonton Symptom Assessment System (ESAS)[32], which is recommended/used in Australian [33] and Canadian [34] cancer services, to aid implementation.

No consensus was reached on the optimal timepoint to commence FCR screening. More participants agreed that screening should commence after treatment completion (67%) than after diagnosis (41%), even though cancer patients (active cancer present) and survivors (no active cancer present) report similar FCR severity [2]. Qualitative feedback from participants acknowledged that patients experience FCR from diagnosis. However, some felt that FCR may be transiently elevated shortly after diagnosis due to uncertainty around treatment outcomes, and asking about FCR at this time may further overwhelm patients. This view conflicts with research showing that health professional-led discussion of recurrence risk/symptoms and related FCR is desired by patients and does not typically trigger higher FCR [6]. The research focused on FCR interventions at the point of diagnosis is lacking, but brief clinician-led interventions involving discussion, normalisation, and validation of FCR in follow-up consultations have demonstrated preliminary efficacy in reducing FCR, with time being the biggest barrier to implementation [6].

Trajectories of FCR from diagnosis appear relatively stable, particularly for patients with high FCR [35, 36], so the timing of initial FCR screening may not greatly affect the level of FCR detected, although screening at treatment completion may help capture clinical FCR (≥ 3 months duration) [3]. Considering these factors, we recommend that FCR screening commence shortly after treatment completion, while acknowledging consensus was not reached. Further research is needed to understand the best time to initiate FCR screening to enable the identification of survivors with FCR who would benefit from intervention and may otherwise be missed.

There was also disagreement regarding the timing of FCR screening and triage conversations relative to routine follow-up care. While screening for FCR before and after follow-up appointments was seen as optimal, participants did not agree screening should be repeated whenever a patient’s clinical management changed, with qualitative feedback reflecting that clinical management may change for varied reasons (e.g. better versus worse disease control). Consensus was also lacking regarding whether triage conversations should ideally occur before or after follow-up scans or appointments. A study of daily FCR levels around routine surveillance scans found those with low baseline FCR showed a similar peak in FCR on the day of surveillance scans to those with high baseline FCR, but FCR levels reduced more slowly and remained elevated post-scan in those with high baseline FCR [37]. Similarly, a scoping review of scanxiety among adults with cancer found that anxiety levels were particularly elevated both before scans and while waiting for results of scans [38]. This suggests having a triage conversation after follow-up scans or appointments where scan results may be delivered could better differentiate between those with temporarily and persistently elevated FCR to guide treatment recommendations. However, further research is needed to support recommendations regarding the optimal timing of triage conversations.

There were some clinical pathway elements that participants agreed certain health professionals were best suited to delivering (e.g. provision of prognostic information by an oncologist). However, FCR was also seen as a concern that all health professionals could play a role in addressing (e.g. through validation and normalisation of FCR). The need for training in conducting FCR screening and triage conversations was broadly endorsed, aligning with previous findings that many health professionals report challenges managing FCR and almost all were interested in training to address FCR [16]. Training oncologists has been found to increase clinician self-efficacy in managing FCR, reduce FCR severity, and be feasible to implement in practice, noting lack of time is an implementation barrier [39, 40]. It was also agreed that with appropriate training, primary care providers could play an important role in addressing mild FCR in routine care. Training on FCR has also been found to increase primary care providers’ knowledge and self-efficacy in identifying and addressing FCR [41] and may help address capacity constraints within the hospital system and the push to deliver more cancer survivorship care in primary care [42]. Integrating FCR training into communication skills training for health professionals managing cancer survivors is likely to aid widespread implementation of the FCR clinical pathway.

Stepped care, where survivors receive universal care regardless of initial FCR levels and are then stepped up to more intensive treatment based on results of rescreening if needed, was endorsed for managing FCR. This contrasts somewhat with recommendations that matched care, where the intensity of intervention is matched to a FCR severity at the outset, may be best [14, 43]. Matched care was not explicitly proposed as an alternative in this study, but the practical application of these two approaches may be similar. Participants agreed that universal care for minimal-mild FCR (e.g. validation and normalisation of FCR) was best delivered by oncologists and nursing staff as part of routine care. In practice, these elements may be integrated into triage conversations, so survivors are likely to receive universal care in both approaches, but more intensive treatment would be delayed until after rescreening in stepped care. One pilot study has shown that matched care is acceptable to survivors, feasible to deliver, and can reduce FCR [44], but further research is needed comparing the implementation of these two approaches on individual and service-level outcomes.

While most proposed items regarding the optimal management of mild and severe FCR reached agreement, there was less agreement, at least initially, around the management of moderate FCR, which affects approximately 40% of people affected by cancer [2]. The proposal that moderate FCR should primarily be managed outside the hospital system and addressed by online or group interventions, which have both shown promising efficacy [8, 45], was not endorsed. Participants also disagreed that non-mental health specialists were the best people to deliver recommended interventions for moderate FCR, instead agreeing that psychologists or allied health (e.g. social workers) are best placed. However, psycho-oncology workforce shortages may necessitate training non-mental health specialists and there was consensus that they could play an important role if trained appropriately. There is a growing literature supporting the acceptability, feasibility, and efficacy of FCR interventions delivered by trained non-mental health specialists, such as primary care providers [41], oncologists [39, 40], and nurses [46, 47]. More interventions that are delivered by non-mental health specialists or online need to be integrated into practice to accommodate the diverse preferences and needs of the large group of survivors with moderate FCR.

Statements suggesting that treatment recommendations should solely be determined by patients’ FCR levels, or conversely by clinician judgements, did not reach agreement. As in the Delphi study developing a clinical pathway for anxiety and depression in adult cancer patients [22], participants acknowledged that it was appropriate for a stepped care algorithm to guide treatment recommendations, but flexibility was needed to tailor the pathway for different populations and service capacities. Clinical pathways have been shown to be most effective in changing practice when there is sufficient flexibility for services to adapt them to their local context [48]. It is critical that the core clinical pathway components (i.e. screening, triage, and treatment) are delivered for optimal FCR care, but decisions around who delivers certain pathway elements and when may be informed by engaging local service stakeholders in process mapping [49, 50] pathway integration into existing workflows.

### Strengths and limitations

A large multidisciplinary sample contributed to the Delphi process, increasing the likelihood that the consensus-based FCR clinical pathway reflects the views of the Australian oncology community, although whether the sample worked with patients across the full range of cancer sites, stages, treatment phases, or survivorship durations is unknown. However, the unknown response rate and limited primary care and surgical oncology representation mean the clinical pathway may not be optimal for addressing FCR in cancer survivors who only have surgical treatment or who are principally managed in primary care, as opposed to in specialist cancer centres. It is unclear why relatively few researchers took part. Some researchers who were collaborators of the study authors may not have participated due to a perceived conflict of interest. There may have consequently been less participants with an in-depth knowledge of the FCR research on which the pathway was based, but a summary was provided to overcome this. While the same group of participants were emailed the survey each round, due to the anonymity of the Delphi responses, the make-up of Round 2 and 3 respondents is unknown and there may have been variability in sample characteristics across rounds.

While the cultural diversity of the Australian oncology workforce is unknown, it is important to note that the study sample did not reflect the cultural diversity of the Australian population more broadly, as no participants were Indigenous and only 12% spoke a language other than English at home. While the study participants themselves were not particularly diverse, it is likely they worked with patients from diverse backgrounds, as reflected in qualitative feedback on cultural and economic considerations relating to some pathway elements. These considerations will be reported in a separate paper on the feasibility of clinical pathway implementation.

This research primarily sought the views of health professionals, who will ultimately be responsible for implementing pathway recommendations in clinical practice; however, future patient input, particularly around items not reaching consensus, such as the timing of FCR screening and triage, would help optimise pathway acceptability. Feedback from culturally and linguistically diverse cancer survivors and health professionals is needed to identify clinical pathway adaptations required to provide culturally sensitive FCR care for different cultural groups, such as Indigenous and minority peoples, who may be differentially affected by FCR and are under-represented FCR research to date [51]. The next phase of this research will explore these perspectives.

The clinical pathway was developed to address FCR in people with a history of early-stage cancer, as that is the population in which most FCR research has been done. However, as noted in the Canadian FCR guidelines [14], there was less supporting evidence for some clinical pathway elements (e.g. timing of FCR screening and treatments for moderate FCR), which seemingly contributed to greater disagreement that could negatively impact on pathway implementation [52]. Further research is needed focused on these areas and on how to identify, assess, and treat fear of progression in the growing numbers of people living with advanced cancer [53], to inform recommendations on management in clinical practice that can be incorporated into future versions of the clinical pathway.

While the FCR clinical pathway was developed in the Australian context, recommendations may apply to similarly developed countries, particularly those with universal healthcare and where most patients are treated in specialist cancer centres. In settings where FCR identification and management are constrained by factors such as limited resources (e.g. developing countries) or insurance coverage (e.g. the USA) [54], the clinical pathway may need to be adapted. This could be done by repeating the Delphi process in those settings (the authors are willing to share all study materials), or the existing pathway could be adapted in partnership with local stakeholders familiar with contextual implementation barriers and enablers and guided by a theoretical framework (e.g. ADAPT-ITT) [55].

### Implications for cancer survivors

This clinical pathway provides clear evidence- and consensus-based recommendations for the optimal management of FCR in clinical practice. Clinical pathway recommendations provide a standardised way for health professionals to optimally identify cancer survivors with FCR, assess severity, determine appropriate treatments, and then deliver or refer for treatment. Implementation of the clinical pathway could significantly reduce the burden of FCR on cancer survivors and the healthcare system.

## Supplementary information

Below is the link to the electronic supplementary material.

Supplementary file1 (PDF 262 KB)

Supplementary file2 (PDF 293 KB)

## Data Availability

The datasets generated and analysed during the current study are available from the corresponding author on reasonable request.
