# Supplementary material for: Step-by-step: A clinical pathway for stepped care management of fear of cancer recurrence—results of a three-round online delphi consensus process with Australian health professionals and researchers
Source: J Cancer Surviv. 2024 Oct 7;20(2):766–80. doi: 10.1007/s11764-024-01685-1 (PMC12988909; doi:10.1007/s11764-024-01685-1)
Supplement: Supplementary file 1 — Supplementary file1 (PDF 262 KB) [file 11764_2024_1685_MOESM1_ESM.pdf]

Step-by-step: A clinical pathway for stepped care management of fear of cancer recurrence– results of a three round online Delphi consensus process with Australian health professionals and researchers

Journal of Cancer Survivorship

Authors
Allan 'Ben' Smith[1], Afaf Girgis[2], Natalie Taylor[3], Alison Pearce[1,4], Jia Liu[5,6,7], Heather L. Shepherd[8], Verena S Wu[1], Gail Garvey[9], Laura Kirsten[10,11], Iman Zakhary[12], Carolyn Ee[13,14], Daniel Ewald[15,16,17], Annie Miller[18], Joanne Shaw[11]

1. The Daffodil Centre, The University of Sydney, A Joint Venture with Cancer Council NSW, Sydney, New South Wales, Australia
2. South West Sydney Clinical Campuses, UNSW Medicine & Health, UNSW Sydney, Australia
3. School of Population Health, UNSW Sydney, Sydney, NSW, Australia
4. Sydney School of Public Health, the University of Sydney, Sydney, New South Wales, Australia
5. St Vincent's Hospital, Sydney, NSW, Australia
6. Faculty of Medicine & Health, UNSW Sydney, Sydney, Australia
7. Garvan Institute of Medical Research, Sydney, NSW, Australia
8. Susan Wakil School of Nursing and Midwifery, Faculty of Medicine and Health, The University of Sydney, New South Wales, Australia
9. The School of Public Health, Faculty of Medicine, The University of Queensland, QLD, Australia
10. Nepean Cancer Services, Nepean Blue Mountains Local Health District, Sydney, NSW, Australia
11. The University of Sydney, School of Psychology, Psycho-Oncology Cooperative Research Group, Sydney, NSW, Australia
12. Multicultural Services, Liverpool Hospital, South Western Sydney Local Health District
13. Caring Futures Institute, Flinders University, Bedford Park, SA, Australia
14. NICM Health Research Institute, Western Sydney University, Penrith, NSW, Australia
15. Lennox Head Medical Centre, NSW, Australia
16. Bullinah Aboriginal Health Service, Ballina, NSW, Australia
17. Sydney University Medical School, Northern Rivers University Centre for Rural Health, NSW, Australia
18. Cancer Council NSW, Woolloomooloo, NSW, Australia

Corresponding Author
Allan 'Ben' Smith
The Daffodil Centre, The University of Sydney, A Joint Venture with Cancer Council NSW, Sydney, New South Wales, Australia
Email: ben.a.smith@sydney.edu.au
Phone: +61 438 634 964

Background

Fear of cancer recurrence (FCR), that is the fear, worry or concern that cancer may return or progress, is very common. According to a large meta-analysis of 11,226 participants from 13 countries, 58.8% of cancer patients and survivors experience moderate to severe FCR, leading to greater distress, poorer quality of life, and greater healthcare use.

There are effective treatments for FCR, but methods to identify those needing help with FCR (i.e. FCR screening) and determine what kind of help may be most suitable (i.e. triage and assessment) are not implemented in routine care. This means that many cancer survivors, defined here as people who have completed active cancer treatment and have no evidence of disease, are missing out on effective FCR treatment. This may explain why help managing FCR is one of cancer survivors most reported unmet supportive care needs.

A team of experts on FCR, health equity and implementation science have developed a draft clinical pathway (i.e., standardised, evidence-based and multidisciplinary plan) for identifying and managing FCR in clinical practice. The draft FCR clinical pathway is based on the scientific literature and similar pathways for anxiety and depression established by the Psycho-Oncology Co-operative Research Group (PoCoG).

Now we need your help to improve and finalise the pathway, which we hope will be useful to, and used by, all Australian cancer services.

What does the pathway look like?

Considering that FCR exists on a continuum from mild to severe we propose that care should be tailored accordingly. The draft clinical pathway constitutes a stepped care model for FCR, where cancer survivors are:

1. <u>Screened</u> for FCR to to identify those who may have clinically significant FCR
2. Engaged in a <u>triage conversation/assessment</u> to confirm the severity of FCR and determine what course of action may be most appropriate
3. Recommended a form of <u>treatment</u> matched with their level of FCR
4. <u>Monitored</u> and stepped up or down as needed.

Rating the pathway

The draft pathway includes suggestions relating to FCR screening, triage and assessment, treatment and monitoring. These suggestions are general, and will probably need to be adapted for individual services. We appreciate that depending on the resources available at your service what is <u>optimal</u> (i.e. likely to achieve the best outcomes for cancer survivors) may be different to what is <u>feasible</u> (i.e. able to be implemented in practice), when it comes to managing FCR.

To aid development of a clinical pathway that can feasibly be implemented now, while also providing an 'ideal' to work towards in future, we are asking you to rate each statement in terms of your agreement with:

1. How <u>optimal</u> the statement is for addressing a cancer survivor's FCR
2. How <u>feasible</u> it would be to implement the statement now

We need a flexible and generalisable pathway that can be adapted easily at the local level. Please keep this in mind when reviewing the proposed pathway statements. On the next page is a figure outlining the pathway for you to look at before you start rating proposed pathway components.

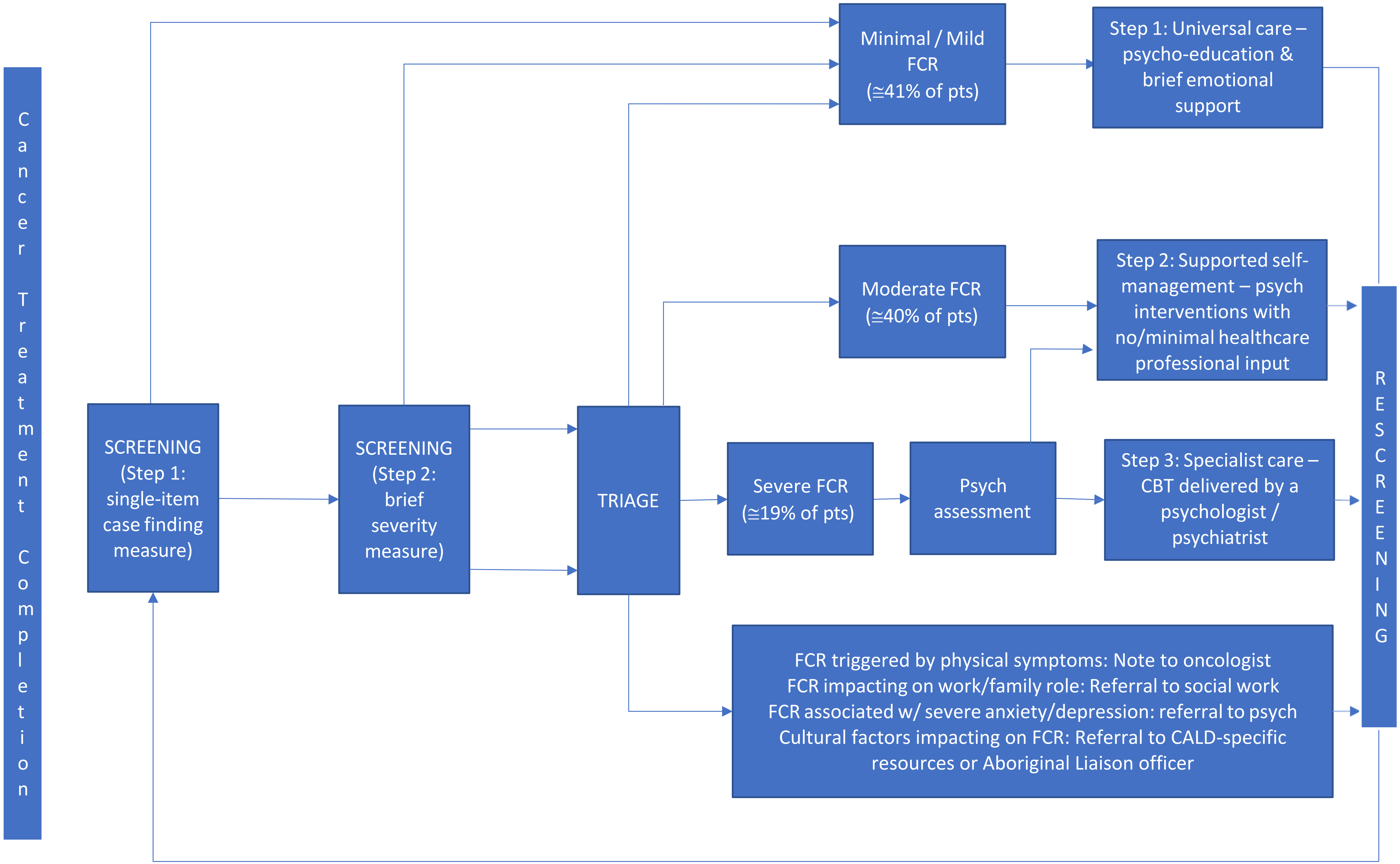
Cancer Treatment Completion
SCREENING (Step 1: single-item case finding measure)
SCREENING (Step 2: brief severity measure)
TRIAGE
Minimal / Mild FCR (≅41% of pts)
Step 1: Universal care – psycho-education & brief emotional support
Moderate FCR (≅40% of pts)
Step 2: Supported self-management – psych interventions with no/minimal healthcare professional input
Severe FCR (≅19% of pts)
Psych assessment
Step 3: Specialist care – CBT delivered by a psychologist / psychiatrist
FCR triggered by physical symptoms: Note to oncologist
FCR impacting on work/family role: Referral to social work
FCR associated w/ severe anxiety/depression: referral to psych
Cultural factors impacting on FCR: Referral to CALD-specific resources or Aboriginal Liaison officer
RESCREENING

Fear of Cancer Recurrence Screening

The first step in the pathway is screening, which refers to the use of brief validated tools, administered either in written or verbal form, to identify cancer survivors who may have clinically significant FCR. Cancer survivors identified through screening would then have a conversation with a healthcare professional to guide referral for further FCR assessment and/or treatment.

What we know: Evidence from screening for anxiety and depression strongly support the use of a two-step process, where people who score above a sensitive cut-off on a very brief / single-item measure then complete a more detailed measure to provide more information about clinical significance. There is emerging evidence indicating that a single-item FCR screening measure (the FCR-1) has good screening performance. There is strong evidence to support the use of the 9-item Fear of Cancer Recurrence Inventory – Short Form (FCRI-SF) in the second step.

What we don't know: There is limited evidence about:

- The best time is to start FCR screening
- The best combination of FCR screening tools
- Who should manage FCR screening
- How often FCR screening should be repeated.

Please indicate your level of agreement about how optimal (i.e., likely to achieve the best outcomes for cancer survivors) and feasible (i.e., able to be implemented in practice) each of the following statements regarding screening for FCR are:

Timing

1. Screening for FCR should commence at the completion of hospital-based treatment (e.g. surgery, chemotherapy or radiotherapy)

	Strongly disagree	Disagree	Neutral	Agree	Strongly agree	Not my expertise	Comments
This statement is optimal	❑	❑	❑	❑	❑	❑	
This statement is feasible	❑	❑	❑	❑	❑	❑	

2. Screening for FCR should be conducted prior to a cancer survivor's follow-up appointment to aid discussion during the appointment if needed

	Strongly disagree	Disagree	Neutral	Agree	Strongly agree	Not my expertise	Comments
This statement is optimal	❑	❑	❑	❑	❑	❑	
This statement is feasible	❑	❑	❑	❑	❑	❑	

3. Screening for FCR should be repeated after a cancer survivor's follow-up appointment, to characterise the persistence of FCR

	Strongly disagree	Disagree	Neutral	Agree	Strongly agree	Not my expertise	Comments
This statement is optimal	❑	❑	❑	❑	❑	❑	
This statement is feasible	❑	❑	❑	❑	❑	❑	
