# Supplementary material for: Step-by-step: A clinical pathway for stepped care management of fear of cancer recurrence—results of a three-round online delphi consensus process with Australian health professionals and researchers
Source: J Cancer Surviv. 2024 Oct 7;20(2):766–80. doi: 10.1007/s11764-024-01685-1 (PMC12988909; doi:10.1007/s11764-024-01685-1)
Supplement: Supplementary file 2 — Supplementary file2 (PDF 293 KB) [file 11764_2024_1685_MOESM2_ESM.pdf]

Step-by-step: A clinical pathway for stepped care management of fear of cancer recurrence– results of a three round online Delphi consensus process with Australian health professionals and researchers

Journal of Cancer Survivorship

Authors
Allan 'Ben' Smith[1], Afaf Girgis[2], Natalie Taylor[3], Alison Pearce[1,4], Jia Liu[5,6,7], Heather L. Shepherd[8], Verena S Wu[1], Gail Garvey[9], Laura Kirsten[10,11], Iman Zakhary[12], Carolyn Ee[13,14], Daniel Ewald[15,16,17], Annie Miller[18], Joanne Shaw[11]

1. The Daffodil Centre, The University of Sydney, A Joint Venture with Cancer Council NSW, Sydney, New South Wales, Australia
2. South West Sydney Clinical Campuses, UNSW Medicine & Health, UNSW Sydney, Australia
3. School of Population Health, UNSW Sydney, Sydney, NSW, Australia
4. Sydney School of Public Health, the University of Sydney, Sydney, New South Wales, Australia
5. St Vincent's Hospital, Sydney, NSW, Australia
6. Faculty of Medicine & Health, UNSW Sydney, Sydney, Australia
7. Garvan Institute of Medical Research, Sydney, NSW, Australia
8. Susan Wakil School of Nursing and Midwifery, Faculty of Medicine and Health, The University of Sydney, New South Wales, Australia
9. The School of Public Health, Faculty of Medicine, The University of Queensland, QLD, Australia
10. Nepean Cancer Services, Nepean Blue Mountains Local Health District, Sydney, NSW, Australia
11. The University of Sydney, School of Psychology, Psycho-Oncology Cooperative Research Group, Sydney, NSW, Australia
12. Multicultural Services, Liverpool Hospital, South Western Sydney Local Health District
13. Caring Futures Institute, Flinders University, Bedford Park, SA, Australia
14. NICM Health Research Institute, Western Sydney University, Penrith, NSW, Australia
15. Lennox Head Medical Centre, NSW, Australia
16. Bullinah Aboriginal Health Service, Ballina, NSW, Australia
17. Sydney University Medical School, Northern Rivers University Centre for Rural Health, NSW, Australia
18. Cancer Council NSW, Woolloomooloo, NSW, Australia

Corresponding Author
Allan 'Ben' Smith
The Daffodil Centre, The University of Sydney, A Joint Venture with Cancer Council NSW, Sydney, New South Wales, Australia
Email: ben.a.smith@sydney.edu.au
Phone: +61 438 634 964

Supplementary Table 1. Content analysis of qualitative feedback on items that did not initially reach consensus.

Pathway component **Item text**	**Consensus level n (%)***	**Category (n)**	**Illustrative quote**
**Round 1**			
1a. Screening for FCR should commence at the completion of hospital-based treatment	63/89 (70.8%)	Fluctuation in FCR levels (n=6)	*"Completion of treatment can be an overwhelming time - recovery from side-effects, fatigue, emotional processing of diagnosis and treatment and this is a time of high normative distress in the weeks to months that follow. FCR may contribute to this distress but may not be primary cause of distress. I find it is in the lead up to the initial scans and follow up that FCR is heightened."*
		Earlier in care continuum (n=6)	*"I believe FCR begins often at diagnosis. Treatment pathway for some patients is very long and this needs to be assessed earlier."*
		Ongoing surveillance needed (n=2)	*"fear starts at the beginning but you should screen at beginning , through treatment and at end of perceived completion of treatment"*
8a. Triage conversations should occur after follow-up scans and appointments, which often trigger heightened FCR	64/86 (74.4%)	Before follow-up (n=6)	*"FCR is highest in the lead up to scans and review appointments - so wouldn't it be better to screen and provide triage conversations in the lead up to scans??"*
		After follow-up (n=2)	*"this is absolutely essential and shows patients that their views are important at each stage of the treatment cycle"*
		Before **and** after follow-up (n=1)	*"In my experience, FCR is much more heightened prior to scans and while waiting for scan results. Patients tend to actually feel relieved after medical appointments. This is coming from a clinical psychologist meeting up with patients before and after medical appointments."*
		Before **or** during follow-up (n=1)	*"Should occur Before or during"*
		During follow-up (n=1)	*"Agree that at first follow-up appointment, or follow-up scans is a good time to screen and triage"*
		Separate to follow-up (n=1)	*"Given the possible transient elevations in distress, I think it would be preferable for these conversations to occur at a time independent of follow-ups (to tease apart clinical FCR from transient distress that has resolved)"*
		At treatment completion (n=1)	*"I wonder if it should occur earlier in the cancer trajectory around the time of treatment completion."*
9a. A triage conversation including discussion of results of FCR screening should take place only with cancer survivors who report moderate or severe FCR	37/86 (43%)	Inclusion of all patients (n=6)	*"A triage conversation about FCR would also benefit those with mild FCR and offer resources and support at that time. FCR is ongoing - and there are periods where it is more pronounced than others; so if we normalise and validate FCR, and equip all patients with knowledge of what it looks like, how to manage it, and where to get support if needed, then hopefully we reduce the risk of patients having severe FCR."*

Pathway component<br>**Item text**	**Consensus level n (%)***	**Category (n)**	**Illustrative quote**
		Inclusion of family and caregivers (n=2)	*" patient or family may also be interested in knowing more"*
		In-depth identification of distress (n=2)	*"yes I think so; otherwise it might be pathologising something that is not causing that much impact/distress"*
		Opportunity for discussion and support (n=2)	*"I believe this is an opportunity to discuss, offer support and navigate"*
16a. All cancer survivors with moderate-severe FCR should be recommended a self-management intervention before being recommended a clinician-delivered intervention	44/84 (52.4%)	Patient-centred approach needed (n=9)	*"I think this should be by patient preference, in combination with triage assessment. Some patients will never take up a seat management intervention."*
		Self-management appropriate (n=3)	*"better to do better to have an intervention while waiting in the queue , it's a positive self empowerment effort and has been shown to assist, if works may reduce the number than onwards needed for a clinical delivered intervention"*
		Clinician-delivered support: moderate-severe FCR (n=3)	*"Moderate-severe FCR is a serious MH [mental health] condition and the effects of distress as well as the effects of the cancer and treatments can negatively impact on patient's ability to interact or engage with the online environment"*
		Dependent on health and English literacy (n=2)	*"this implies a high level of health literacy (and general literacy), as well as English language proficiency to be self-managing."*
		Dependent on wait times (n=1)	*"based on the wait times to access support"*
		Support for borderline moderate-severe FCR (n=1)	*"potential gap for those who are borderline moderate-severe"*
17a. The specific FCR treatment recommended to a cancer survivor will be determined by individual clinicians	56/83 (67.5%)	Patient input needed (n=6)	*"Cancer survivors should be included in this decision making"*
		Clinician decision (n=3)	*"I think there should be guidance from the screening algorithms, but that individual clinicians may be able to override it based on additional clinical information they may have"*
		Expert referral (n=2)	*"it should be determined by the screening tool and an expert in [FCR]"*
		Differs according to sites and clinicians (n=1)	*"As long as a pathway is followed - may not be viewed similarly across sites/clinicians"*
		Depends on step (n=1)	*"Will depend on the Step"*
		Clear pathway guidelines needed (n=1)	*"To an extent. But the guideline should be clear for less experienced staff."*
18a. If a cancer survivor does not accept the recommended FCR intervention, an alternative intervention within the same step should be	66/84 (78.6%)	Further assessment (n=1)	*"If the person is not agreeable then a conversation and further assessment around this should be made as to an appropriate and acceptable course of action that they are agreeable to."*
		Individualised approach needed (n=1)	*"interventions have to be individualised - not determined by a step up or down. Clinicians have to be trained in a range of interventions, including third wave*

Pathway component **Item text**	**Consensus level n (%)***	**Category (n)**	**Illustrative quote**
suggested, then interventions from the step below			*interventions, alternative therapies, adjunct /complimentary therapies and not just a cognitive or behavioural approach"*
		Patient input (n=1)	*"If Patient is agreeable."*
		Uncertainty over monitoring (n=1)	*"not sure how this would be monitored?"*
24a. Primary healthcare professionals (e.g., GPs or GP nurses) are the ideal people to address mild FCR as part of routine care	51/81 (63%)	Lack of knowledge and expertise (n=4)	*"GPs don't have the specialist knowledge but could perform screening assessments for FCR and referral onward to treating team"*
		Training for GPs (n=2)	*"I find that a good GP is central to providing ongoing care and support to patients. They may need training to attend to this though."*
		Cost considerations for patient (n=1)	*"risk and FCR is not linear , GPs are great - how many survivors have to pay to see their GP's now though"*
		Less reliance on GP (n=1)	*"Patients may not put as much faith in GPs..."*
		Mental health professionals – mild FCR (n=1)	*"Social Workers and MH social workers, MH OT's are well positioned to address FCR. Many of these clinicians work within GP clinics and are competent to address FCR and other symptoms of psychological distress"*
27a. If cancer survivors report minimal/mild FCR in rescreening, then no further action is needed	29/80 (36.3%)	Ongoing surveillance needed (n=8)	*"I would say they should be monitored on an ongoing basis."*
		Support options still needed (n=4)	*"Maybe information about where and how to access further support if needed but also re-screening may be warranted at a later date in case it does become relevant to some people."*
		Determined by patient preference and profile (n=2)	*"It would be great if it was a choice for the patient however this would make the system more complex"*
28a. Moderate FCR should be addressed by online or group psychological interventions	45/79 (57.0%)	Depends on patient choice (n=4)	*"not everyone is comfortable in group settings"*
		Limited feasibility (n=4)	*"It is a good option to have for the general population but it will not appeal to some population groups (elderly, CALD, Indigenous, Low socio-economic access)."*
		Offers social support (n=1)	*"well its practical and they often derive peer support"*
		Support via other platforms (n=1)	*"It can be, but other interventions/platforms too"*
29a. Moderate FCR should primarily be addressed outside the hospital setting	37/77 (48.1%)	Support accessibility (n=3)	*"Patients have reported that they prefer to have their concerns addressed outside the treatment centre. Rural and remote patients describe intense fatigue, travelling large distances and inconvenience of therapeutic interventions when coinciding with treatment can be difficult and exhausting"*
		Care continuity (n=2)	*"If there is shared info and communication between two sites (outside hospital and treating hospital)"*

Pathway component<br>**Item text**	**Consensus level n (%)***	**Category (n)**	**Illustrative quote**
		Hospital setting (n=2)	*"by who exactly? More expertise within health system"*
		Hospital visits triggering (n=2)	*"hospitals can strongly trigger FCR; smells, returning to treatment facilities, staff, and even driving in the direction of hospitals where treatment was given can be big triggers for FCR."*
		Cancer Council (n=1)	*"Somewhere like Cancer Councils could do this; but not sure re primary health"*
		Depends on patient circumstances (n=1)	*"It depends. Some come to the hospital a lot for ongoing surveillance and (if they are on active treatment for example hormone therapy). I would say this group should see a psych in the hospital."*
		Experienced staff (n=1)	*"best addressed by staff who are competent and knowledgeable in FCR"*
		Off-site FCR support to be clarified (n=1)	*"that would be nice but where?"*
30a. Non-mental health specialists (e.g. oncology nurses) are the best people to deliver the interventions recommended	26/78 (33.3%)	Dependent on experience (n=3)	*"My experience is that such nurses, through well trained in oncology and medical /treatment options and treatments, do not have the necessary skills or knowledge to deliver effective MH [mental health] interventions. To do so, is to minimise the psychological impacts of FCR as not being a legitimate MH concern"*
		Further training needed (n=3)	*"Could be, with assistance from appropriate trained mental health clinicians ([social worker] or psych or mental health trained nurse)"*
		Mental health specialists appropriate (n=2)	*"Think the evidence suggests that mental health specialists are still the best people, just also among the most costly!"*
		Cancer care co-ordinators appropriate (n=2)	*"CNC [cancer nurse co-ordinator] yes – RN [registered nurse] on floor no"*
		Funding and resource considerations (n=2)	*"they do a lot of other things too but yes have the continuity and trust of the patient and family, how are more to be funded though?"*
		Limited time and capacity (n=1)	*"Best positioned but maybe not the best profession. Many professionals within the team would be equally placed and skilled. It's just that the cancer nurses are most central. Do they have time/capacity?"*
32a. Healthcare professional-delivered booster sessions should be offered to patients whose FCR is improved but not remitted at rescreening	62/79 (78.5%)	Feasibility uncertain (n=2)	*"unsure whether it is possible to incorporate this level of monitoring in large populations of patients"*
		Depends on patient need (n=1)	*"If people want this."*
		Established rapport essential (n=1)	*"Surely the person who delivered the initial Tx [treatment] would be the preferred option as developed Tx rapport etc"*
		Ongoing FCR surveillance (n=1)	*"In principle, I agree, but also see value in monitoring to see if improvements continue to develop over time as patients continue to implement strategies"*

Pathway component<br>**Item text**	**Consensus level n (%)***	**Category (n)**	**Illustrative quote**
**Round 2**			
1a. Screening should commence at the completion of hospital-based treatment (e.g. surgery, chemotherapy or radiotherapy)	46/69 (66.7%)	After treatment completion (n=5)	"*At the completion of treatment patients often feel left alone and may have an increase FCR*"
		Towards treatment completion (n=3)	*"In my experience it's as people are finishing up treatment and heading home that FCR begins to be discussed"*
		Fluctuation in FCR levels – later (n=2)	*"There is so much variation in treatment regimes, type and order that a one size fits all approach does not seem optimal to me. However, at the end of treatment, after high focus on "just getting through" there is often a drop in mood along with time to think about recurrence."*
		Before treatment completion (n=2)	"*FCR begins before treatment is completed. Usually after chemo and before [radiotherapy].*"
		At treatment completion (n=1)	"*My main concern here is that defining "completion" is not always easy to define, but on the assumption there is clarity, I agree*"
		At diagnosis and throughout treatment (n=1)	*"It is not always clear to patients or perhaps professionals involved with a patient when treatment is finalised as often there are plans for ongoing screening &/or treatment. Sometimes FCR is present at diagnosis and could be well supported throughout the treatment trajectory."*
		At diagnosis (n=1)	*"should be at diagnosis (especially given the definition of FCR includes progression, so applies to advanced/incurable [cancer])"*
		Later after treatment (n=1)	*"too early"*
1b. Screening for FCR should commence shortly after diagnosis of cancer	28/68 (41.2%)	Timing not appropriate (n=6)	*" Patients just received news they got cancer and needs to undergo treatment, why you discourage them by saying " by the way your cancer might comes back. Please fill up this form on how you feel about that". Patient might freak out. Its a BIG NO from me."*
		After diagnosis (n=2)	*"Needs to be identified within treatment to the person for monitoring and support"*
		Depends on disease/treatment stage (n=2)	*"Not all patients need to be screened for FCR. Those with early stage disease for whom treatment is time limited and straightforward may not experience FCR. Those who do will still be able to be identified at follow up."*
		Heightened FCR at diagnosis (n=1)	*"FCR would be elevated at diagnosis"*
		In conjunction with other screening (n=1)	*"Are we assuming other screening such as distress screening is taking place?"*

Pathway component<br>**Item text**	**Consensus level n (%)***	**Category (n)**	**Illustrative quote**
3b. Screening for FCR should be repeated whenever changes are made to a patient's clinical management (e.g., initiation of new treatment or change in follow-up frequency)	52/68 (76.5%)	Repeat screening after changes to clinical management (n=2)	*"Important to check understanding and if new/extra support required"*
		No repeat screening (n=1)	*"no"*
		After treatment completion (n=1)	*"They starting a second line of treatment, they now their cancer back. Maybe screen them after second line of treatment finishes."*
		During or after treatment completion (n=1)	*"If already assessed for an indicated early in a treatment process it can then be scheduled for review throughout treatment or another screening conducted upon completion of treatment."*
8a. Triage conversations should occur <u>after</u> follow-up scans and appointments, which often trigger heightened FCR	43/68 (63.2%)	After follow-up (n=7)	*"Triage conversations should occur after scans if the screening for fear of recurrence rates high or the scan results create a high level of distress in a patient"*
		Timing not appropriate (n=2)	*"Scan appointments can elicit FCR in many who have little fear at other times, this does not seem the appropriate time to capture. FCR can also occur with pain and other changes in health."*
		Before follow-up (n=1)	*"I would agree that it might be useful to hold triage conversations before the appt to help manage that common fear. Whether that level found then is representative of their general level is another question. To sample their real FCR, I would tend to sample a month after."*
8b. Triage conversations should occur <u>before</u> follow-up scans and	43/68 (63.2%)	Before follow-up (n=2)	*"Triage conversations should occur only if the pre scan screening indicates a high level of fear"*

Pathway component **Item text**	**Consensus level n (%)***	**Category (n)**	**Illustrative quote**
appointments, to capture, normalise and help survivors manage heightened FCR at this time		Before follow-up - earlier (n=1)	*"A number of patients practice avoidance, and this may cause distress. Putting plans in place before this time would be great (so not the week before the appointment)"*
		After follow-up (n=1)	*"Patient will be very anxious waiting for the results. Why not wait until they got the scan result and they more calm and collected."*
		Timing dependent on patient (n=1)	*"Timing needs individual clinical assessment, not prescriptive timing"*
		Timing not appropriate (n=1)	*"This is probably the time of the most heightened "scanxiety" as treatment has stopped and more time to ruminate. But I don't believe this captures FCR, just typical anxiety"*
9a. A triage conversation including discussion of results of FCR screening should take place only with cancer survivors who report moderate or severe FCR	15/68 (22.1%)	Inclusion of all patients (n=5)	*"Regardless the outcome, all patients needs to know what kind of help available for them, when they needed"*
		Depends on healthcare professional (n=1)	*"Knowledge is power, and knowledge can cause distress. I just don't feel I know enough/there is sufficient information for me to rank this. I also feel how it is done and by who will make a big difference to whether it should be done"*
		Screening unnecessary if triage conducted with all (n=1)	*"If you are going to have triage conversations with all, what is the point of screening? However, we then need to be clear that our screening measures are optimal and have good sensitivity and specificity for clinically significant levels of FCR"*
16a. All cancer survivors with moderate-severe FCR should be recommended a self-management intervention before being recommended a clinician-delivered intervention	36/66 (54.5%)	Offer both options (n=3)	*"Although it would be ideal to offer a combination of both self-management interventions and a clinician-delivered intervention if there is capacity for this"*
		Patient-centred approach (n=3)	*"I think this is too prescriptive. some patients may not be able to participate in self-management. this includes patients with diverse abilities, and cognitive impairments, e.g. brain cancer patients."*
		Self-management offered during wait time (n=1)	*"self-management whilst waiting to be seen, but no to be re-triaged."*
		Stepped care approach appropriate (n=1)	*"Best practice, but may not be achievable"*
17a. The specific FCR treatment recommended to a cancer survivor will be determined by individual clinicians	28/66 (42.4%)	Depends on capacity (=2)	*"Capacity differs across Services. This should certainly be considered as allowed within a stepped-care approach"*
		Awareness of individual important (n=1)	*"Knowledge of the person is more important than the decision of an individual clinician. Other health professionals may be more aware than a single clinician."*
		Clinician decision (n=1)	*"Clinicians should follow a recommended treatment pathway or algorithm but use their assessment skills and knowledge of local/available resources to identify which interventions on the pathway are feasible for each individual patient."*

Pathway component **Item text**	**Consensus level n (%)***	**Category (n)**	**Illustrative quote**
		Limited expertise in FCR (n=1)	*"Clinicians are not well educated in FCR (the majority)"*
		Patient input needed (n=1)	*"If options available discussion with cancer survivor on their preference"*
27a. If cancer survivors report minimal/mild FCR in rescreening, then no further action is needed	17/66 (25.8%)	Ongoing monitoring needed (n=5)	*"FCR could change, so rescreening at various timepoints should be carried out ideally"*
		Support options needed (n=2)	*"Provide information on how to self-manage or where help is available IF worsens."*
		Incorporate in routine care (n=1)	*"If it's a very quick and easy process, it's probably easier to keep it as a routine task at certain reviews/time intervals than to keep track of when the last screening occurred and whether it needs to be repeated or not, etc"*
		Risk of under-reporting FCR (n=1)	*"Self report may not always reflect patient experience. The tendency to please the doctor can result in under-reporting."*
		Clarification what "no further action" means (n=1)	*"It depends what no further action is required means, you would still want them to know their prognosis, signs of recurrence etc., and if you are screening would you not still screen?"*
28a. Moderate FCR should be addressed by online or group psychological interventions	28/64 (43.8%)	Patient choice ideal (n=3)	*"Choice is important"*
		Need more support options (n=3)	*"It's an option but it shouldn't be the only option."*
		Limits access for some patients (n=2)	*"This limits access for CALD folk or people with low IT literacy or access"*
		Individual interventions (n=1)	*"individual is better"*
		Concerns about validity of interventions (n=1)	*"Ultimately, but in the above a range of evidence-based and not yet validated approaches are combined, which is very worrying."*
29a. Moderate FCR should primarily be addressed outside the hospital setting	22/65 (33.8%)	Hospital setting (n=3)	*"If a Pt is continuing to attend the hospital setting for review appointments, it would be better to keep this in the hospital setting"*
		Both settings (n=1)	*"It can be addressed in both"*
		Community support (n=1)	*"There is a role for NGO / cancer specific community based groups top run these e.g. cancer Council / leukaemia Foundation / Lymphoma Australia etc."*
		Experienced staff (n=1)	*" requires clinicians with expert knowledge of cancer"*
		Hospital visits triggering (n=1)	*"Hospital can bring trauma of having cancer treatment"*
		Incompatible with external settings (n=1)	*"Doubtful the corresponding support services exist to support this strategy"*
		Support accessibility (n=1)	*"depending where support services are situated"*

Pathway component<br>**Item text**	**Consensus level n (%)***	**Category (n)**	**Illustrative quote**
		Off-site FCR support to be clarified (n=1)	*"I think this would be ideal but in reality who would be addressing it then?"*
30b. With further training, non-mental health specialists (e.g., oncology nurses), could play an important role in delivering interventions recommended for moderate FCR	49/65 (75.4%)	Dependent on experience and awareness (n=1)	*"It is the oncology experience that is necessary here to understand the issues combined with mental health awareness"*
		Limited expertise (n=1)	*"Expertise dubious."*
		Non-mental health specialists (n=1)	*"gives the best chance of covering more patients"*
32b. Booster sessions should be offered to patients whose FCR is improved but not remitted after being rescreened once or more, ideally with the healthcare professional who delivered the initial intervention	51/65 (78.5%)	Delivered by anyone (n=1)	*"Should be with anyone"*
**Round 3**			
1a. Screening for FCR should commence at the completion of hospital-based treatment (e.g. surgery, chemotherapy or radiotherapy)	52/72 (72.2%)	After treatment completion (n=3)	*"This would provide a consistent approach, whereas delaying screening for "a few weeks" may mean loss of some patients."*
		Approaching treatment completion (n=2)	*"Should commence as approaching completion. Patients struggle with FCR from day of diagnosis."*
		Flexibility needed over timing (n=2)	*"There needs to be flexibility over the timing for different tumour types that might have very short hospital encounters or prolonged hospital encounters."*
		Before treatment completion (n=1)	*"I would like to see screening occur prior to the end of hospital based treatment"*
		During treatment (n=1)	*"during treatment"*
		Part of treatment completion (n=1)	*"I can see the earlier/later rationales but I wonder if we could just say that FCR screening should be part of the completion of hospital-based treatment"*
3b. Screening for FCR should be repeated whenever changes are made to a patient's clinical management (e.g., initiation of new treatment or change in follow-up frequency)	57/73 (78.1%)	Repeat screening after changes to clinical management (n=3)	*"We need to recognise that our patients are not unaware of potential consequences or implications, and at times of changes to a patient's management, very likely to stimulate or exacerbate FCR"*
		Limited feasibility in practice (n=2)	*"I cannot imagine it is practical. And I am not sure if those prompts are necessarily the ones that would precipitate anxiety in most pts."*
		Depends on patient circumstances (n=2)	*"Like many clinical decisions, this has to be made to a large extent on an individual basis, when there is a change in clinical management, it most often signifies a deterioration, and patients know this. To suddenly then ask "are you afraid that the cancer will come back" at this point could be insensitive. Assessment for FCR remains*

Pathway component **Item text**	**Consensus level n (%)***	**Category (n)**	**Illustrative quote**
			very important, but patients need some time to adjust to changes, before we then assess where they have now landed and feel about it all in themselves."
		Pairing screening with treatment not appropriate (n=1)	*"The problem with pairing screening with changes in treatment is that these are times when FCR might be artificially inflated."*
8a. Triage conversations should occur after follow-up scans and appointments, which often trigger heightened FCR	50/72 (69.4%)	After follow-up (n=5)	*"Screening before a scan is akin to taking an oral temperature immediately after your patient has finished a hot cup of tea, better to recognise they will be fearful beforehand, and then to assess afterwards, regardless of the findings of the scan."*
		Before follow-up (n=2)	*"I feel this is okay, but would probably be more useful before the scans that heighten FCR"*
		Uncertainty about timing (n=2)	*"how soon after?"*
		Timing not appropriate (n=1)	*"screening at this time is likely to identify elevated FCR not representative of their usual state. Or conversely if after scan results reduction in FCR due to good results that is only temporary and may increase again a few weeks later. Treatment for managing temporary FCR around scans is also different from managing pervasive FCR"*
